# Drug Intake and Actinic Keratosis: A Case-Control Study

**DOI:** 10.5826/dpc.1102a31

**Published:** 2021-04-12

**Authors:** Andrea Sechi, Ambra di Altobrando, Eugenio Cerciello, Elisa Maietti, Annalisa Patrizi, Francesco Savoia

**Affiliations:** 1 Dermatology, IRCCS Policlinico di Sant’Orsola. Department of Experimental, Diagnostic and Specialty Medicine, Alma Mater Studiorum University of Bologna, Italy; 2 Department of Biomedical and Neuromotor Sciences, University of Bologna, Italy

**Keywords:** drug reaction, photosensitivity, actinic keratosis, angiotensin receptor blockers, antiplatelet drug

## Abstract

**Background:**

Actinic keratosis (AK) is a form of premalignant keratinocyte dysplasia. Recently, the role of photosensitizing drugs in the development of AK has been postulated.

**Objective:**

This study evaluated a possible association between the use of photosensitizing drugs and the development of AK. A secondary aim was to identify a possible association between any medication other than those primarily examined and AK.

**Methods:**

A single-center, case-control study assessed the cumulative drug exposure of 90 patients with AK and 90 controls visiting a dermatology service for other skin ailments. Before the visit, patients were interviewed to collect data on daily therapy and the lag-time of discontinued drugs within the last 2 years, and to record the drug’s active ingredient, dosage, and duration of therapy. In addition, sociodemographic characteristics including age, sex, educational level, skin phototype, and cumulative sun exposure habits were gathered.

**Results:**

By logistic regression, exposures to angiotensin II receptor blockers (ARBs) and antiplatelet agents were identified as independent risk factors for the development of AK. ARB intake was associated with AK only at high exposure (OR = 13.6; 95% CI, 2.0–93.8). The use of antiplatelet drugs was borderline, yet not significant, at low exposure (OR = 3.31; 95% CI, 0.86–12.7), but increased in a dose-dependent manner. The strongest correlation was found at the highest cumulative dose (>1100 dose unit-years (OR = 4.38; 95% CI, 1.16–16.6).

**Conclusions:**

High exposure to ARBs and antiplatelet agents may promote AK carcinogenesis in at-risk patients.

## Introduction

Actinic keratosis (AK) is a common skin lesion caused by the proliferation of atypical keratinocytes. In the literature, AK is considered as a form of either premalignant dysplasia or in situ squamous cell carcinoma (SCC). The finding that 60% of SCCs arise at the site of a previous AK supports the pathogenetic model of a continuous progression between AK and SCC [1]. AKs may evolve following 1 of 3 paths: spontaneous regression, stable existence, or malignant progression. Natural remission positively correlates with working outdoors due to decreased sun sensitivity, and has been observed in more than 50% of AKs. However, regressed lesions easily reappear over time [2].

The risk of malignant transformation of AK into an invasive SCC has been investigated in many studies. A 0.6% progression rate for a single AK was reported over 1 year, and 2.6% in 4 years [1]; in a 20-year period, this rate ranges from 0.025% to 20% [2]. The reason for such a wide difference is mainly the lack of a consensus definition of AK, with no risk stratification according to AK features. AK clinical presentation is highly polymorphic, with a variable combination of erythema, hyperkeratosis, and telangiectasia. In this scenario, AKs with rapid growth, bleeding, ulceration, induration and inflammation, and those >1 cm in size are at increased risk of progression to invasive SCC [3].

There is extensive evidence regarding AK risk factors. For instance, UV irradiation promotes carcinogenesis through the induction of p53 mutations, which elude cell-cycle checkpoints and promote the uncontrolled proliferation of dysplastic keratinocytes [2]. Recent advances confirm the influential role of UVB rays in the formation of thymidine dimers in the DNA and RNA of keratinocytes [2]. As a consequence, cumulative sun exposure is closely related to the onset of AK and SCC in the photo-exposed areas [4]. Both a history of sunburns, mainly those occurring in childhood [5], and chronic sun exposure (due to outdoor occupations or hobbies) increase the risk of developing AK, with an increasing trend towards older ages [4]. Moreover, the most susceptible subjects have fair skin phototypes [4] or are immunosuppressed (eg, transplant recipients and people with genetic disorders affecting DNA-repair mechanisms) [2]. Additional risk factors are male sex, a smoking habit, and the assumption of photosensitizing drugs [6,7]. The assumption of drugs that target the skin by any mechanism (eg, dermal deposit, skin thinning), such as oral retinoids, topical retinoids, tetracycline, macrolides, aminoquinolines, amiodarone, and methoxypsoralen, make the skin more vulnerable to sun damage [7].

In 2018, the European Medicines Agency (EMA) released a warning concerning the risk of developing non-melanoma skin cancer (NMSC) from the use of thiazide diuretics [8]. The EMA Pharmacovigilance Risk Assessment Committee based this warning on the results of a Danish case-control study that found dose-response relationships between the long-term intake of hydrochlorothiazide and the risk of developing basal cell carcinoma and SCC [9]. Therefore, patients assuming hydrochlorothiazide alone or in combination with other diuretics, or nondiuretic antihypertensives, especially those with prior NMSC, are recommended to apply sunscreen daily and to attend regular dermatological check-ups. Prompted by this warning, we performed a prospective, single-center case-control study to evaluate a possible association between the use of photosensitizing drugs and the development of AK. A secondary aim of the study was to identify a possible association between any medication other than those primarily examined and AK.

## Materials and Methods

Over a 6-month period, 90 AK cases were recruited consecutively from the dedicated NMSC Outpatient Service and 90 controls were recruited from several services of general dermatology at the Sant’Orsola-Malpighi Hospital. The study was approved by Central Emilia Wide Area Ethical Committee; it started on January 1, 2020, and ended on May 31, 2020.

A cut-off age higher than 60 years was set as an inclusion criterion for both groups. A medical history of AK or NMSC was an exclusion criterion for controls. All patients provided written informed consent for enrollment in the study.

To collect information on sociodemographic characteristics, skin phototype, cumulative sun exposure, and drug intake, patients were interviewed on a standardized questionnaire and then examined by an experienced dermatologist. Sun exposure was considered high in the case of more than a 5-year outdoor occupation or more than 10 years of outdoor recreational exposure. The survey also inquired about the long-term use of any class of medications, including drugs taken for at least 2 consecutive years and withdrawn within 3 months of the index date. Data recorded included the drug’s active ingredient, daily dosage, duration of therapy and, in case of dosage variation, the new dosage and year of variation. For each patient, cumulative exposure was calculated as the product of the duration of treatment multiplied by the daily dosage (dose unit-years).

## Statistical Analysis

Continuous data were described by means and standard deviations (SD) or by median and interquartile ranges in case of a strongly asymmetric distribution (eg, exposure to drugs). For each categorical variable, we reported the absolute and relative frequencies expressed as percentages.

The comparison between cases and controls was performed using the t-test for continuous variables with a normal distribution (eg, age) and the Mann-Whitney test for continuous variables with a skewed distribution (eg, drug exposure). Pearson’s chi-square test and Fisher’s exact test were used for comparisons of categorical variables. To assess an association between drug exposure and AK development, we used a multiple logistic regression model adjusted for the possible confounding effects of sociodemographic characteristics. The results of logistic regression were expressed as odds ratios (ORs) and confidence intervals (95% CI).

Statistical significance was set at P = .05. The analyses were conducted using the statistical software Stata version 15 (StataCorp, College Station, TX, USA).

## Results

Overall, 180 patients were assessed, 90 cases and 90 controls (Table 1). The 2 groups differed significantly in age, sex, skin type, and recreational sun exposure (P < .001). Compared with controls, cases were older (mean, 79.4 vs. 73.3 years), more frequently male (73.3% vs. 38.9%), and more likely to have skin phototype I–II (52.2% vs. 14.4%). Combined sun exposure was lacking in 11.1% of cases and 46.6% of controls, mild-moderate in 4.4% and 18.9%, and high in 84.4% and 34.4%, respectively. No difference in terms of educational level was observed between the groups.

Drug exposure differed between groups for 2 classes of medications: angiotensin receptor blockers (ARBs) and antiplatelet drugs (Table 2). Similar proportions of patients took ARBs in the case and control groups (22.2% vs. 18.9%); however, the cumulative exposure was significantly higher in cases (P < .015). On the other hand, more than twice as many cases than controls took antiplatelet drugs (48.9% vs. 22.2%). No association was found between AK and other drugs, including thiazides and other diuretics, statins, ß-blockers, calcium channel blockers, ACE inhibitors, anticoagulants (warfarin and novel oral anticoagulants) and the antiarrhythmic drug amiodarone (data not shown).

The exposure to ARBs and antiplatelet agents was categorized into 3 classes: high, low, and no exposure. Cut-off values of 750 and 1100 unit-years, respectively for ARBs and antiplatelet drugs, were used to distinguish high vs. low exposure. These new variables were included in a logistic regression model, adjusted for age, sex, skin type, and sun exposure (Table 3). Even after adjusting for sociodemographic features, the exposure to ARBs and antiplatelet agents was significantly associated with the presence of AK. An increase in the probability of AK was found only with high exposure to ARBs (OR = 13.6; 95% CI, 2.0–93.8). The low exposure to antiplatelet agents was not significant at the statistical analysis (OR = 3.31; 95% CI, 0.86–12.7), but the high exposure was significant (OR = 4.38; 95% CI, 1.16–16.60), showing a dose-dependent association.

Eight cases were concomitantly exposed to ARBs and antiplatelet drugs, whereas no double exposure was found in controls (Table 4).

## Discussion

Two studies based on Danish national data showed a dose-dependent, cumulative association between the photosensitizing activity of hydrochlorothiazide and NMSC, including basal cell carcinoma and SCC [9,10]. One of these studies highlighted a 7-fold increased risk of SCC lip cancer in exposed subjects [10]. The other study found the strongest associations with: a cumulative hydrochlorothiazide dose >200 mg, female sex, upper or lower limbs, and age <50 years [9]. The postulated pathogenetic mechanism is photosensitivity due to hydrochlorothiazide, which is well documented and could enhance photocarcinogenesis [1–14].

Subsequently, further papers on the association between photosensitizing agents and NMSC were published and yielded conflicting results. Indeed, some studies showed no association between hydrochlorothiazide and NMSC, and some authors raised concerns about the methodological limitations of the 2 Danish studies [15–24].

In contrast to the Danish studies, our study did not find any evidence of an association between thiazide diuretics and actinic keratosis, probably due to the limited use of thiazides in our sample. Instead, our study showed that high exposure to ARBs and even low exposure to antiplatelet drugs, in a dose-dependent pattern, represent significant risk factors for the development of AK.

In the literature, a similar study conducted in 1999 assessed the impact of photosensitizing drugs on the development of AK. However, the number of patients was small (34 cases and 34 controls), and the use of cardiovascular drugs was also considerably low [7].

Other recent studies demonstrated the induction of photosensitivity in patients taking ARBs, and hypothesized drug-driven carcinogenesis favored by sun exposure [25–29]. However, as for hydrochlorothiazide, the results of different studies are contradictory. Conflicting evidence regarding the use of antiplatelet agents comes from a Brazilian case-control study, which demonstrated a protective effect of acetylsalicylic acid on the development of AK [30]. This study enrolled 74 cases and 216 controls, and the median duration of therapy was 36 months. The use of acetylsalicylic acid was an independent protective factor for AK onset since it was associated with a lower AK count on the face and upper extremities, regardless of concomitant risk factors. In our study, ticlopidine and clopidogrel were used by only 3 cases and 1 control: for this reason, we included all antiplatelet agents within a single group.

The main limitation of our study is that it is inadequately powered to detected mild differences in drug exposures between cases and controls. For instance, the differential percentage in the use of oral diuretic thiazides (22.2% vs. 13.3%) would require a sample size of 288 cases and 288 controls to be significant at P < .05, with 80% power.

Furthermore, cases were not closely matched to controls in terms of age, sex, or skin phototype. Consecutive recruitment of patients was pursued to avoid selection bias but implied a significant inhomogeneity in the 2 groups. The study design foresaw these results, and aimed to adjust all possible confounding factors by multivariate analysis.

In southern Europe, the high prevalence of AK made the recruitment of controls extremely difficult in older age groups. Besides, the case group had significantly more men and fair-skin individuals. The sex difference between the groups may have affected their use of sun-protective measures, as women are more inclined to apply sunscreen. However, sun-protective behaviors were not recorded in our survey. They could have affected the quality of our data due to the dose-dependent ability of sun-blockers to increase the AK remission rate and prevent the development of new lesions [2].

Another limitation of our study is the inclusion of patients using multidrug therapies, which, however, reflects real practices and the disease burden of elderly patients. The current study was underpowered to identify potential synergic or antagonistic effects of the simultaneous use of multiple drugs. Supplementary analyses adjusted for the concomitant use of antihypertensive/diuretic drugs should be performed in a larger series. A possible synergistic effect of taking two photosensitizing drugs favoring AK genesis could not be excluded. In our study, the number of patients on both antiplatelet and ARB therapy was very low. Table 4 shows that none of the controls with high exposure to antiplatelet agents was exposed to ARBs at the same time. In contrast, the 8 patients on dual therapy with high exposure to antiplatelet drugs and ARBs were in the case group. Therefore, a more extensive study with a larger population size is warranted to characterize patients taking both drugs, and to define the attributable risk of developing AK for each medication.

## Conclusions

Our study was designed to investigate a possible association of photosensitizing drugs and AK. Our results suggest a correlation between the use of ARBs or antiplatelet agents and the onset of AK. ARBs possess a known photosensitizing effect, which could be the missing link between their use and the development of AK. Antiplatelet agents do not induce photosensitivity, and their association with AK remains unknown. Further studies are needed to assess the pro-carcinogenic potentials of these 2 classes of drugs, in order to prompt adequate dermatological screening and sun protection, mainly in patients with a medical history of NMSC.

## Figures and Tables

**Table 1:** Sociodemographic Features of Cases and Controls

Factor	Cases (*n* = 90)	Controls (*n* = 90)	*P*
Age, mean ± SD	79.4 ± 7.2	73.3 ± 8.6	<.001

Sex, No. (%)			<.001
Male	66 (73.3)	35 (38.9)	
Female	24 (26.7)	55(61.1)	

Education, No. (%)			.103
Primary school	37 (41.1)	26 (28.9)	
Middle school	19 (21.1)	18 (20.0)	
High school	23 (25.6)	23 (25.6)	
University degree	11 (12.2)	23 (25.6)	

Skin phototype, No. (%)			<.001
I–II	47 (52.2)	13 (14.4)	
III–IV	43 (47.8)	77 (85.6)	

Sun exposure at work, No. (%)	12 (13.3)	5 (5.6)	.07

Recreational sun exposure, No. (%)			<.001
Absent	16 (17.8)	45 (50.0)	
Mild-moderate	4 (4.4)	17 (18.9)	
High	70 (77.8)	28 (31.1)	

Combined sun exposure, No. (%)			<0.001
Absent	10 (11.1)	42 (46.7)	
Mild-moderate	4 (4.4)	17 (18.9)	
High	76 (84.4)	31 (34.4)	

**Table 2:** Frequency and Duration of Exposurea to the Examined Drugs

Drug Class	Statistical Parameter	Cases (*n* = 90)	Controls (*n* = 90)	*p*
Angiotensin receptor blockers	n (%)	20 (22.2)	17 (18.9)	.580
	Exposure	960 [469–2360]	300 [90–750]	.015
Antiplatelet drugs	n (%)	44 (48.9)	20 (22.2)	<.001
	Exposure	1100 [750–1780]	750[300–1215]	.037
Statins	n (%)	29 (32.2)	30 (33.3)	.874
	Exposure	110 [80–180]	100 [60 – 190]	.382
Calcium channel blockers	n (%)	15 (16.7)	18 (20.0)	.563
	Exposure	60 [35–100]	80 [40–240]	.638
Beta-blockers	n (%)	29 (32.2)	24 (26.7)	.414
	Exposure	400 [60–1100]	97.5 [20–860]	.133
ACE inhibitors	n (%)	29 (32.2)	29 (32.2)	1.0
	Exposure	52.5 [45–120]	55 [27.5–180]	.870
Warfarin	n (%)	7 (7.8)	8 (8.9)	.787
	Exposure	45 [20–115]	50 [22.5–97.5]	1.0
Novel oral anticoagulants	n (%)	2 (2.2)	5 (5.6)	.444
	Exposure	10 [5–15]	75 [40–110]	.171
Thiazides	n (%)	20 (22.2)	12 (13.3)	.119
	Exposure	225 [87.5–412.5]	137.5 [56.25–387.5]	.402
Loop diuretics	n (%)	17 (18.9)	15 (16.7)	.697
	Exposure	165 [50–250]	150 [50–400]	.406

Drug exposure was calculated as the daily dose multiplied by the years of therapy (dosage unit-years) and is reported as the median (interquartile range).

ACE = angiotensin-converting enzyme.

**Table 3:** Multiple Logistic Regression Model Predicting Actinic Keratosis

Variables	No.	OR (95% CI)

ARBs		
• No exposure	143	1.00
• Low exposure	18	0.25 (0.05 – 1.32)
• High exposure (>750 dose unit-years)	19	13.6 (2.0 – 93.8)

Antiplatelet agents		
• No exposure	116	1.00
• Low exposure	31	3.31 (0.86 – 12.69)
• High exposure (>1100 dose unit-years)	33	4.38 (1.16 – 16.60)

Note: the model is adjusted for age, sex, skin type, and sun exposure.

ARBs = angiotensin receptor blockers; CI = confidence interval; OR = odds ratio.

**Table 4:** Concomitant Exposure to ARBs and Antiplatelet Drugs

	Cases				Controls			
	Exposure to Antiplatelet Drugs, n (%)				Exposure to Antiplatelet Drugs, n (%)			
	Low	Moderate	High		Absent	Low	High	
**Exposure to ARBs**	Low	38 (42.2)	16 (17.8)	16 (17.8)	Absent	58 (64.4)	8 (8.9)	7 (7.8)
	Moderate	3 (3.3)	1 (1.1)	2 (2.2)	Low	8 (8.9)	4 (4.4)	0 (0)
	High	5 (5.6)	1 (1.1)	8 (8.9)	High	4 (4.4)	1 (1.1)	0 (0)

ARBs = angiotensin receptor blockers.
